# Internet addiction among adolescents during the COVID-19 pandemic: Associations with sociodemographic and psychological distress

**DOI:** 10.7717/peerj.17489

**Published:** 2024-06-28

**Authors:** AyuZeity Bistari Md Bukhori, Mohd Hasni Ja’afar

**Affiliations:** Department of Public Health Medicine, Universiti Kebangsaan Malaysia, Cheras, Kuala Lumpur, Malaysia

**Keywords:** Adolescent, COVID-19, Internet addiction, Internet, Behavior, Psychological symptoms

## Abstract

**Background:**

The COVID-19 pandemic has had tremendous implications for billions of adolescents worldwide due to school closures, forcing students to embrace internet usage for daily tasks. Uncontrolled use of the internet among adolescents makes them vulnerable to internet addiction (IA). This study aims to determine the prevalence of IA among adolescents and assess its association with sociodemographic factors, smartphone use, and psychological distress during the pandemic.

**Method:**

A cross-sectional self-administered online survey was conducted among students aged 13–17 from May 15^th^, 2021, until May 30^th^, 2021, using the Malay version of the Internet Addiction Test (MVIAT), the Depression, Anxiety, and Stress Scale (DASS-21), and the Coronavirus Impacts Questionnaires, as well as a sociodemographic information form. The data was analyzed with IBM SPSS Statistics version 23.

**Results:**

A total of 420 adolescents participated in the survey. The majority of them (70.7%) were female, with a mean age of 15.47 years (±1.49 years old). About 45.5% of the respondents were classified as internet addicted users. The Chi-square test analysis showed that age (*p* = 0.002), smartphone usage (*p* = 0.010), rate of midnight use (*p* < 0.001), frequency (*p* < 0.001), duration (*p* < 0.001) of device usage, and presence of depression, anxiety, and stress (p < 0.001) were all significantly associated with IA. Multiple logistic regression showed age (aOR = 1.16, 95% CI [1.00–1.35], *p* = 0.048), smartphone usage (aOR =3.52, 95% CI [1.43–8.67], *p* = 0.006), mild or moderate depression (aOR = 2.43, 95% CI [1.36–4.34], *p* = 0.003), severe or extremely severe stress (aOR = 6.41, 95% CI [2.18–18.82], *p* = 0.001) were significantly related to IA.

**Conclusions:**

Late adolescence, the use of smartphones, and the presence of psychological distress like depression, and stress were potentially associated with IA. Wise use of smartphones and early identification of any psychological distress among adolescents are warranted, especially during the pandemic.

## Introduction

As COVID-19-related concerns appear to be gradually diminishing on a global scale (World Health Organization, 2023), comprehensive examination of how pandemic-related experiences have influenced and continue to shape various aspects of human behavior remains essential. Despite the easing of immediate health worries, the long-lasting effects and adaptations stemming from the pandemic necessitate ongoing scrutiny. The COVID-19 pandemic has devastating implications towards education institutions that impacted around 1.6 billion students worldwide due to school closures, forcing students to embrace internet usage in their daily tasks (Jason & John, 2020). Nonetheless, increased internet exposure puts an adolescent at risk of developing internet addiction (IA) and related psychological impacts that yet need to be explored (Lai et al., 2015).

IA is characterized as poorly controlled or excessive preoccupations with internet use, which is associated with tolerance development and withdrawal symptoms (Ooi et al., 2020). Numerous addictive behavior patterns such as smoking, excessive drinking, and IA are expected to increase in the current pandemic situation, as has been observed in previous disaster events such as natural catastrophes, war conflict, and the severe acute respiratory syndrome (SARS) epidemic (Dimaggio, Galea & Li, 2009; Lin, 2020). In contrast to adult populations, adolescents are particularly susceptible to internet preoccupation. This vulnerability arises from their consistent and repetitive exposure to various online attractions, including social media, shopping platforms, games, unlimited movie access, and other enticing online activities (Vannucci et al., 2020). The diverse and easily accessible nature of these internet-based attractions led to multiple negative outcomes that potentially ruined job and school performance, family function, and life satisfaction (Lai et al., 2015; Li et al., 2019).

When addressing adolescents who display symptoms of IA, Zhao et al. (2023) emphasized the significance of raising concern regarding possible coexisting psychological conditions. This is important as one of the etiologies of IA is considered to be due to the psychopathological dimensions’ existence preceding the behavioral symptoms of IA (Dimaggio, Galea & Li, 2009). Experiencing severe depressive and anxiety symptoms before COVID-19 was found to significantly predict an increased tendency for IA (Zhao et al., 2023) and positively predict Internet Game Disorder severity and videogame use (Teng et al., 2021) during the pandemic. In addition, concurrent and significant associations were observed between internet addiction (Lee et al., 2008) and depressive disorders (Wrase et al., 2006) *via* “short” alleles located in the serotonin-transporter-linked promoter region (5-HTTLPR). The possibility exists that serotonin dysfunction could be linked to vulnerabilities in both depressive symptoms and internet addiction, as suggested by the close association between the 5-HTTLPR gene and serotonin function (Lee et al., 2008).These findings align with previous studies conducted on adolescent psychiatric patients, indicating that excessive internet usage may induce abnormal alterations in brain regions and systems. Specifically, these changes involve the prefrontal cortex and limbic system, which play crucial roles in regulating behavioral and emotional control (Cai et al., 2023; Cerniglia et al., 2017). Numerous factors, including fear of virus infection, family health status, family income instability, and future uncertainty as a sequela of lockdown, have contributed to the development of stress and emotional disturbances among adolescents (Shah et al., 2020).

As countries endeavor to manage and mitigate the impacts of the pandemic, the increasing prevalence of IA is recognized as a significant mental health concern that requires careful attention and proactive intervention. Malaysia has a diverse and multicultural society with distinct cultural norms and values. Understanding IA within the Malaysian cultural context allows for the development of interventions and strategies that are culturally sensitive and tailored to the unique needs of Malaysian adolescents. Cultural factors can significantly influence how adolescents engage with the internet. Studying IA in the context of Malaysian culture helps identify cultural nuances that may impact internet use patterns, preferences, and the potential risks associated with excessive online activities. Findings from studies on IA among Malaysian adolescents can inform the development of policies and prevention programs. A culturally informed approach ensures that these initiatives resonate with the values and norms of Malaysian society.

The main objective of this population-based study is to determine the prevalence of IA and identify its associated risk factors among secondary school adolescents in the Southeastern state of Malaysia during the ongoing pandemic. The study will address the following key research questions (RQs) and hypotheses (H):

RQ 1: What are the sociodemographic factors associated with an increased risk of IA during the COVID-19 pandemic?

H1: It is hypothesized that older adolescents, male gender, lower education level, lower status of parent’s education level, and lower median monthly family income are more prone associated with IA.

RQ 2: Do perceived COVID-19 impacts (financial, resource, and psychological) have a meaningful association with IA as compared to normal internet users?

H2: It is hypothesized that the perceived COVID-19 impact score is significantly associated with IA.

RQ 3: Is the presence of psychological distress in adolescence (depression, anxiety, and stress) during the COVID-19 epidemic positively related to IA?

H3: It is hypothesized that depression, anxiety, and stress are positively related to IA.

## Materials & Methods

### Research design

This study used a cross-sectional design and utilized a simple random sampling technique to select secondary schools in the Melaka districts that are under the administration of the Ministry of Education.

### Participant

The study participants comprise students aged 13 to 17 years. Participation is voluntary, and electronic consent is obtained from parents or guardians through an anonymous, self-administered online questionnaire. Adolescents who are confirmed by their parents to be suffering from brain diseases (such as seizures) and psychiatric diseases or are undergoing psychiatric treatment and adolescents from Special Education Classes are excluded from the study. Responses lacking completeness in online questionnaires are deemed invalid and are excluded from the analysis.

**Data collection tools** MVIAT is used to measure the presence of IA that is described elsewhere (Young, 2015). The questionnaire contains 20 items graded on a five-point Likert scale that examine several patterns of symptoms over the last 30 days, including salience, excessive use, neglect of work, anticipation, lack of control, and neglect of social life. The maximum total achievable is 100, with a cutoff point of 43. Any result in excess of 43 classifies an individual as IA (Chong Guan et al., 2015). The MVIAT has been validated in Bahasa Melayu by Chong Guan et al. (2015) and has good internal consistency (Cronbach’s alpha = 0.91), parallel reliability (intra-class coefficient correlation = 0.88, *p* < 0.001) and concurrent validity with the Compulsive Internet Use Scale (Pearson’s correlation = 0.84, *p* < 0.00) (Chong Guan et al., 2015).

The M-DASS-21 version was used to assess the level of depression, anxiety, and stress symptoms in the reference period of the past one week. It consists of 21 items graded by a 4-point Likert scale, with each domain surveyed (depression, anxiety, stress) comprising seven questions, and the highest graded score is 21. Questions numbers 3, 5, 10, 13, 16, 17, and 21 represented depression subscales that were divided into people that do not have depression (0–5), mild (6–7), moderate (8–10), severe (11–14), and extremely severe (15 and more). Questions numbers 2, 4, 7, 9, 15, 19, and 20 represented anxiety subscales, which were divided into people that do not have anxiety (0–4), mild (5–6), moderate (7–8), severe (9–10), and extremely severe (11 and more). Questions numbers 1, 6, 8, 11, 12, 14, and 18 represented stress subscales that were divided into people that do not have stress (0–7), mild (8–9), moderate (10–13), severe (14–17), and extremely severe (18 and more). The M-DASS-21 version has been translated by Osman et al. (2014) and the validity obtained Cronbach’s alpha values of (Depression = 0.84; Anxiety = 0.74; and Stress = 0.79) respectively.

The Coronavirus Impacts Questionnaires were used to assess individuals’ experiences with the COVID-19 disease and how it had affected their lives, including on a financial scale, resource scale, and psychological scale. The questionnaire consists of 6 items and the answers are graded on a 7-point Likert scale. The scale is designed to be used in a continuous fashion, and averages for each scale for each participant will be derived by statistical analysis. The Coronavirus Impacts Questionnaire (Short) has been developed by (Conway, Woodard & Zubrod, 2020) and the questionnaire performed well in Confirmatory Factor Analyses, suggesting that it can be utilized as a separate coherent questionnaire set (CFI = .95, TLI = .93, RMSE = .10, *p* < .001). The short version 6-item scale also yielded high internal reliability for each component (Financial = .76, Resource = .93, Psychological = .89) that showed a similar range as such long version questionnaire (Conway, Woodard & Zubrod, 2020).

### Procedure

The questionnaire was distributed through representative teachers at selected schools in tandem with social media and WhatsApp telecommunication platforms for optimal coverage. Respondents were required to answer a set of questionnaires containing five major domains, starting with sociodemographic background followed by habitual internet use questions. The remaining three domain questions are based on the Malay Version Internet Addiction Test (MVIAT), the Malay Version Depression, Anxiety, and Stress Scale-21 (M-DASS-21), and the Coronavirus Impacts Questionnaires, all of which the authors have obtained permission to use from the respective copyright holders.

### Statistical analysis

The data was analyzed using IBM SPSS Statistics version 23 (SPSS Inc., Armonk, NY, USA). Descriptive data is presented as frequency and percentage. Demographic characteristics, habitual internet use, level of depression, anxiety, and stress of respondents with and without IA were compared by using the Chi-square test. An independent-sample *t*-test was used to compare the mean of the Coronavirus Impacts scores between the types of internet users. Simple and multiple logistic regression analyses were performed to observe the relationship between the studied factors and IA. A *p*-value of less than 0.05 was regarded as statistically significant in this study.

### Ethics

The study was approved by both the Ethical Committee Board, Faculty of Medicine, Universiti Kebangsaan Malaysia (UKM) (Reference no: UKM PPI/111/8/JEP-2021-336) and the Educational Policy Planning and Research Division, Ministry of Education (MOE) Malaysia.

## Results

The total enrollment included 420 adolescents aged 13–17 years old (184 from Melaka Tengah district, 146 from Alor Gajah district, and 91 from Jasin district). The overall mean age of the participants was 15.47 years old (±1.49 years old) with male respondents being younger (14.84 ± 1.58 years old) than females (15.78 ± 1.37 years old). The majority of the respondents were female (*n* = 297; 70.7%) compared to male adolescents (*n* = 123; 29.3%).

### Prevalence of IA, NIU and demographic characteristics

The mean score for the total MVIAT was 43.49 ± 12.59 (Male, 43.26 ± 11.78; Female, 43.59 ± 12.93) (Table 1). Based on the total MVIAT score, 45.5% (male = 43.9%; female = 46.1%) of the respondents were classified as having IA while the remainder, 54.5% (male = 56.1%; female = 53.9%), were considered normal internet users (NIU). The mean IAT score in the IA group was 54.65 ± 9.34, while in the NIU was 34.18 ± 5.21. There was a significant difference between age and education status among IA and NIU (*p* < 0.05). Meanwhile, there were no statistically significant differences in the rates of gender, ethnicity, being an only child, mother and father’s education level, and median monthly family income among both groups. Thus, the first hypothesis was only partially accepted.

**Table 1:** Comparison of demographic characteristics and habitual internet use among internet addicts and normal internet users.

**Sociodemographic** **Factors**	**Internet addiction user** **n = 191**	**Normal internet user** **n = 229**	***x*** ^**2**^	***p*** **value**
Age, *n (%)*				
13-year-old	26 (13.6)	42 (18.3)	16.575	0.002^*^
14-year-old	14 (7.3)	43 (18.8)		
15-year-old	21 (11.0)	23 (10.0)		
16-year-old	47 (24.6)	51 (22.3)		
17-year-old	83 (43.5)	70 (30.6)		
Gender, *n (%)*				
Male	54 (28.3)	69 (30.1)	0.174	0.677
Female	137 (71.7)	160 (69.9)		
Ethnicity, *n (%)*				
Malay	159 (83.2)	191 (83.4)	0.048	0.976
Chinese	21 (11.0)	24 (10.5)		
Indian & Others	11 (5.8)	14 (6.1)		
Education status, *n (%)*				
Lower Secondary Form (Form 1–3)	61 (31.9)	108 (47.2)	10.038	0.002^*^
Higher Secondary Form (Form 4–5)	130 (68.1)	121 (52.8)		
The only child, *n (%)*				
Yes	19 (9.9)	21 (9.2)	0.073	0.787
No	172 (90.1)	208 (90.8)		
Mother’s education level				
Primary school	10 (5.2)	8 (3.5)	1.712	0.887
Secondary school	83 (43.5)	107 (46.7)		
Pre-university	34 (17.8)	41 (17.9)		
Degree	44 (23.0)	47 (20.5)		
Master/PhD	9 (4.7)	14 (6.1)		
Others	11 (5.8)	12 (5.2)		
Father’s education level				
Primary school	6 (3.1)	11 (4.8)	4.446	0.487
Secondary school	98 (51.3)	123 (53.7)		
Pre-university	31 (16.2)	28 (12.2)		
Degree	32 (16.8)	37 (16.2)		
Master/PhD	17 (8.9)	15 (6.6)		
Others	7 (3.7)	15 (6.6)		
Median Monthly Family income				
B40	105 (55.0)	134 (58.5)	1.073	0.585
M40	62 (32.5)	73 (31.9)		
T20	24 (12.6)	22 (9.6)		
**Habitual internet use**				
Type of electronic device				
Smartphone/Tablet	182 (95.3)	202 (88.2)	6.658	0.010^*^
Laptop/Desktop/Notebook	9 (4.7)	27 (11.8)		
Frequency, *n (%)*				
>2 times/day	164 (85.9)	164 (71.6)	21.595	<0.001^*^
4–6 times/week	25 (13.1)	37 (16.2)		
1–3 times/week and No use	2 (1.0)	28 (12.2)		
Frequency of use after 00:00, *n (%)*				
>4 times/week	99 (51.8)	40 (17.5)	67.030	<0.001^*^
>3 times/week	28 (14.7)	30 (13.1)		
Twice/week	25 (13.1)	45 (19.7)		
Once/week	18 (9.4)	35 (15.3)		
No use	21 (11.0)	79 (34.5)		
Duration per day, *n (%)*				
>6 h	95 (49.7)	56 (24.5)	44.784	<0.001^*^
4–6 h	58 (30.4)	60 (26.2)		
2–4 h	26 (13.6)	69 (30.1)		
<2 h	12 (6.3)	44 (19.2)		

**Notes.**

Pearson’s Chi-square test.

* Significant *p* values (*p* < 0.01).

### Habitual internet use among IA and NIU

Smartphones or tablets were found to be the most favorable electronic devices (IA = 95.3%; NIU = 88.2%), followed by laptops, desktops, and notebooks (IA = 4.7%; NIU = 11.8%). The frequency and duration of internet use, as well as the use of electronic devices after midnight (00:00 h), were all significantly higher in IA than in the NIU group (*p* < 0.001) (Table 1).

### Prevalence of depression, anxiety, and stress among IA and NIU

Depression, anxiety, and stress were found to have an overall prevalence of 38.4% (*n* = 161), 45.0% (*n* = 189), and 35.2% *(n* = 148), respectively. Table 2 shows that the prevalence of different levels of depression, anxiety, and stress was significantly higher in the IA group than in the NIU group (*p* < 0.001). Therefore, the third hypothesis was supported.

**Table 2:** Prevalence of depression, anxiety, and stress among internet addicts and normal internet users.

**DASS-21 Class**	**Internet addiction** **user** **n = 191**	**Normal internet user** **n = 229**	***x*** ^**2**^	***p-*** **value**
Depression, *n (%)*				
Severe and extremely severe	53 (27.7)	25 (10.9)	49.193	<0.001^*^
Mild and moderate	55 (28.8)	28 (12.2)		
No	83 (43.5)	176 (76.9)		
Anxiety, *n (%)*				
Severe and extremely severe	57 (29.8)	27 (11.8)	43.839	<0.001^*^
Mild and moderate	62 (32.5)	43 (18.8)		
No	72 (37.7)	159 (69.4)		
Stress, *n (%)*				
Severe and extremely severe	22 (11.5)	7 (3.1)	66.487	<0.001^*^
Mild and moderate	85 (44.5)	34 (14.8)		
No	84 (44.0)	188 (82.1)		

**Notes.**

Pearson’s Chi-square test.

* Significant *p* values (*p* < 0.01).

### The mean difference of impact COVID-19 values

Interestingly, there was no significant difference in COVID-19 Impact values manifested by the IA group in comparison with the NIU group in terms of Impact Financial of 3.43 (±1.65) as compared to 3.14 (±1.69) in the NIU (*p* = 0.079), Impact Resource (IA = 2.84(±1.70); NIU = 2.66 (±1.57) and *p* = 0.246), and Impact Psychological (IA = 3.72 (±1.92); NIU = 3.66 (±1.77) and *p* = 0.755) (Table 3). Hence, the second hypothesis was not supported.

**Table 3:** Comparison of impact COVID-19 mean score with internet users class.

	**Internet addiction user** **n=191**		**Normal internet user** **n=229**		***t***		***p-*** **value**	
	**Mean**	**(SD)**	**Mean**	**(SD)**			
**Impact COVID-19** ^**a**^							
Impact financial	3.43	1.65	3.14	1.69	−1.763		0.079
Impact resource	2.84	1.70	2.66	1.57	−1.16		0.246
Impact psychological	3.72	1.92	3.66	1.77	−0.31		0.755

**Notes.**

Independent t-test.

### Association between age, smartphone, psychological symptoms, COVID-19 impacts with IA

Table 4 shows the multiple logistic regression analysis results of the factors involved in the development of IA. Age, depression, stress, and smartphones were identified as significant associated factors with IA. The odds of IA in older adolescents were 1.16 times higher than in younger adolescents (aOR = 1.16, 95% CI [1.00–1.35], *p* = 0.048). The odds of IA in adolescents with mild or moderate depression domains of the DASS-21 score were 2.43 times higher than those who had a normal score (aOR = 2.43, 95% CI [1.36–4.34], *p* = 0.003). The odds of IA in adolescents with severe or extremely severe stress domains of the DASS-21 score were 6.41 times higher than those who had a normal score (aOR = 6.41, 95% CI [2.18–18.82], *p* = 0.001). Adolescents who use smartphones or tablets were 3.52 times more likely to develop IA as compared to those using other devices (aOR = 3.52, 95% CI [1.43–8.67], *p* = 0.006).

**Table 4:** Factors associated with internet addiction by using multiple logistic regression analysis.

Variables	**Crude OR**	95% CI	Wald	*p* value	Adjusted OR	95% CI	Wald	*p* value
Age	1.25	(1.10, 1.43)	11.07	0.001	1.16	1.00, 1.35	3.90	0.048
								
Depression			46.73	<0.001			21.30	<0.001
Mild to moderate	4.17	(2.47, 7.04)	28.42	<0.001	5.62	2.63, 11.76	20.92	<0.001
Severe to extremely severe	4.50	(2.61, 7.73)	29.49	<0.001	0.80	0.16, 4.01	0.07	0.789
								
Stress			61.16	<0.001			25.70	<0.001
Mild to moderate	5.60	(3.48, 8.99)	50.77	<0.001	9.71	3.96, 23.82	24.67	<0.001
Severe to extremely severe	7.03	(2.89, 17.10)	18.52	<0.001	4.60	0.40, 52.23	1.51	0.219
								
Anxiety			41.89	<0.001	–	–	–	–
Mild to moderate	3.18	1.97, 5.14	22.52	<0.001	–	–	–	–
Severe to extremely severe	4.66	2.73, 7.97	31.70	<0.001	–	–	–	–
								
COVID-19 impact financial	1.11	(0.99, 1.24)	3.08	0.079	–	–	–	–
COVID-19 impact resource	1.07	(0.95, 1.21)	1.35	0.246	–	–	–	–

**Notes.**

Multiple logistic regression analysis.

* Significant *p* values (*p* < 0.05).

** Significant *p* values (*p* < 0.01).

## Discussion

In the midst of the COVID-19 pandemic, the significance of understanding the intricate relationship between IA and psychological distress has become more pronounced, particularly in the context of Malaysia. The current surge in internet usage, although crucial for sustaining connectivity during periods of physical isolation, gives rise to significant concerns regarding the potential consequences of increased internet usage on mental well-being. Exploring the association between IA and psychological distress within the Malaysian population is imperative, not only for addressing the immediate challenges posed by the pandemic but also for fostering a resilient and psychologically sound society in the post-pandemic era.

### Prevalence of IA among adolescents

Pre-pandemic studies using similar diagnostic tools of IAT revealed that the overall prevalence of IA ranged from 2.6% in Northern and Western Europe to 10.9% in Middle Eastern countries (Cheng & Li, 2014). Meanwhile, the prevalence of IA was between 1.0 and 9.0% among children and adolescents in Europe, 1.0 to 12.0% in the Middle East, and 2.0 to 18.0% in Asia (Christakis, 2010). Local data in Malaysia, however, demonstrated a higher range of IA, ranging from 2.4% to 49.2% (Abdul Aziz et al., 2018; Lai et al., 2015). Specifically, in the region of Malacca, the incidence of IA stands at 29.4% (Ministry of Health Malaysia, 2017). This indicates a substantial variation in IA rates within the country, emphasizing the importance of localized studies to capture the nuances of internet usage patterns and associated addictive behaviors. Understanding the prevalence of IA at the regional level, such as in Malacca, contributes to more targeted and effective interventions to address the specific needs of the local population in managing and preventing internet-related addictive behaviors.

Meanwhile, recent studies conducted amid the pandemic indicate a notable rise in the occurrence of IA compared to pre-pandemic rates, with figures ranging between 24.4% and 46.8% in various countries (Dong et al., 2020; Lin, 2020; Priego-Parra et al., 2020; Sun et al., 2020). The present study adds to the increasing amount of evidence by demonstrating that 45.5% of the respondents were classified as addicted users. Interestingly, this surge in IA during the pandemic aligns with historical patterns observed in previous disaster events such as during the severe acute respiratory syndrome (SARS) epidemic, natural disasters, and war (Dimaggio, Galea & Li, 2009; Lin, 2020).

The surge in IA during the COVID-19 pandemic can be attributed to several interconnected factors. Firstly, the finding that the lockdown period had a higher prevalence rate of overall behavioral addiction (*e.g.*, internet addiction, gaming addiction, gambling addiction, smartphone addiction, social media addiction) than the non-lockdown period suggests that the advancement of the internet and individuals’ coping strategies during the lockdown played significant roles. Specifically, the lockdown measures may have heightened individuals’ psychological distress, prompting some to resort to potentially addictive behaviors as coping mechanisms (Alimoradi et al., 2022). Additionally, the closure of schools and the shift to online learning due to COVID-19 have transformed adolescents’ educational experiences, resulting in heightened use of digital devices and increased online activities (Besalti & Satıcı, 2022; Kusnadi & Kaloeti, 2022). With limited outdoor activities and reduced face-to-face interactions, adolescents have redirected their focus to online pursuits, encompassing social networking, online gaming, entertainment and educational tasks (Onukwuli et al., 2023; Patel, Patel & Patel, 2022; Putra, Fithriyah & Zahra, 2023; Woon, Daud & Razak, 2021).

### Age and IA

The finding has shown that the occurrence of IA increased with age, as seen in the current study population aged 13 (13.6%) as compared with aged 17 (43.5%). The findings of this study’s regression analysis indicated a statistically significant association between age and internet addiction, with the likelihood of being classified as an addictive internet user increasing by 16% for each additional unit in age. (aOR = 1.16, 95% CI [1.00–1.35], *p* = 0.048). This result aligns with the observations made in seven European countries and Taiwan, which also indicate that the prevalence of dysfunctional internet behavior is greater among adolescents aged 16–17 in comparison to those aged 14–15 (Lin, 2020; Tsitsika et al., 2014). Malaysia, on the other hand, has described the prevalence of IA as increasing by 38% among 13 to 17 years old students (Ministry of Health Malaysia, 2018), and (Ooi et al., 2020) suggested that age is a significant predictor of IA as the prevalence would increase with the increasing age of the user. One plausible explanation for the observed association between age and addictive internet use may be attributed to the considerable academic pressure and studying load faced by older students in preparation for a more established future career pathway; thus, older adolescents utilize the internet more than younger ones to ensure better understanding and efficacy of learning (Chen et al., 2022; Hu et al., 2021).

### Gender and IA

Gender, on the other hand, has revealed no specific sex-related predominantity in this current finding when compared to other previous literature. The possible reasons for the gender difference observed are different online activities, duration of online surfing, and dissimilar self-control and emotional regulation between boys and girls that distinguish the two groups’ proneness to become addicted to the internet. (Dong et al., 2020; Liu et al., 2011) also emphasized the gender-linked pattern, suggested that girl’s maturity reflects a greater understanding of their actions and consequences, resulting in a higher prevalence of IA among female respondents.

A dissimilar pattern seen in the present study reflects equal habitual online activities in both genders in terms of types of electronic devices used, frequency of electronic device use, hours of surfing the internet, and frequency of online activity after midnight. Moreover, it is worth noting that these habitual internet use factors were all significantly higher among IA groups than among NIU groups. As such, the pandemic situation altered the pattern of online surfing due to a variety of factors, including various psychological effects and stress, a high level of virtual support, and lower functioning families experienced by adolescents. These factors all contributed significantly to the prediction of IA (Lin, 2020).

### IA and psychological symptoms

Research indicates a reciprocal relationship between addiction behaviors, such as internet addiction, and mental symptoms (Afifi et al., 2016). Psychiatric symptoms and disorders can instigate addictive behaviors, while engaging in addictive behaviors can also elevate the risk of experiencing psychiatric symptoms and disorders (Cai et al., 2023). The current study unveiled those individuals in the IA groups showed significantly higher levels of depression, anxiety, and stress during assessments compared to those in the NIU group. From the regression analysis, mild or moderate depression (aOR = 2.43, 95% CI [1.36–4.34], *p* = 0.003), (OR = 4.50, *p* < 0.001), severe or extremely severe stress (aOR = 6.41, 95% CI [2.18–18.82], *p* = 0.001) (OR = 7.03, *p* = 0.001), and anxiety (OR = 4.66, *p* < 0.001) were significantly related with IA. Indeed, the confirmation of a significant positive relationship between depression, anxiety, and stress with IA in our analysis aligns with numerous prior studies in the literature. An abundance of empirical research, spanning from cross-sectional studies (Abdul Aziz et al., 2018; Dong et al., 2011; Lai et al., 2015; Li et al., 2019) to longitudinal research, strongly supports the notion that increased time spent on internet either smartphones, social media, or gaming has not only worsened psychological distress (Chen et al., 2021; Chen et al., 2022), but pre-existing psychological distress also significantly and positively predicts IA (Zhao et al., 2023) and positively predicted internet Gaming Disorder severity (Teng et al., 2021).

Primary school students in China have documented notably elevated levels of psychological distress throughout the COVID-19 pandemic in comparison to the period preceding the outbreak due to their increased time spent on internet-related activities (Chen et al., 2021). The increased psychological distress that was noted during the pandemic period may be ascribed to the concerning rates of COVID-19 transmission and mortality that were reported (Ahorsu et al., 2020; Chen et al., 2021). As people contend with the continuous cascade of distressing news and the concrete consequences of the virus on their localities, emotions of apprehension, dread, and unpredictability inherently heighten. Adolescents were vulnerable to the overabundance of infodemic and negative feelings, such as fear and worries that were constantly spread and expressed on social media. In due course, the information obtained may intensify stress responses, heightened anxiety, and depression, increase their concern towards the situation, resulting in media consumption and further distress. This cycle is vicious and difficult to break, as the internet is always applicable, and information readiness is able to feed the curiosity enquired by adolescents (Biedroń et al. 2021; Priego-Parra et al., 2020). Meanwhile, Hu et al. (2021) suggested that IA among adolescents was the result of stressors induced by the pandemic situation as a maladaptive coping mechanism to overcome these negative emotions, which had to occur *via* the presence of a second mediator: learning burnout. Learning burnout develops when adolescents are kept socially distant at home due to fear of COVID-19 transmission, thus increasing online engagement that leads to negative effects on their academic performance and daily learning process. Subsequently, it promotes emotional disturbance among the vulnerable (Hu et al., 2021).

The current investigation nevertheless failed to establish a significant association between pandemic stressors, including impact financial, impact resource, and impact psychological with IA. This finding suggests that, in the context of the examined population, the heightened stressors brought about by the pandemic may not directly contribute to an increased likelihood of developing IA. Several factors could contribute to this lack of significant association. It’s possible that other individual, social, or environmental factors play a more prominent role in the development of IA in this specific population, mitigating the impact of pandemic-related stressors. Additionally, coping mechanisms, resilience, and pre-existing conditions may influence how individuals respond to stressors and their susceptibility to IA.

Our multivariate analysis confirmed that the use of smartphones or tablets has greater odds of IA. The types of devices are usually related to the likelihood of a certain individual remaining online, either for academic purposes or recreational use. According to Dong et al. (2020), the primary internet devices used among internet-addicted users (90.9%) during the COVID-19 pandemic were smartphones and tablets. Although desktop computers are commonly used by students for online learning, their inclination towards smartphones is frequently influenced by an extensive array of user-friendly attributes. Young students find smartphones to be an appealing and practical educational aid due to their inherent portability, accessibility, and the wide variety of learning applications that are available to them (Chen et al., 2021; Uther & Ylinen, 2018). Pre-pandemically, consistent findings yielded locally have shown smartphones as the device of choice for internet access, particularly adolescents, and the wide availability of electronic devices was reported to have significantly contributed to the soaring number of IA (Ooi et al., 2020).

The advantage of this study came from its considerably large sample size, involving 420 respondents. Furthermore, the direct focus of this study on the prevalence of IA and its associated factors at the time of the COVID-19 pandemic has supplemented new knowledge and information, particularly in the context of adolescence in this country. Nevertheless, several limitations highlighted above can be used as added value in future related studies. Firstly, this study was only conducted among adolescents in Malacca state, which means it could not represent the large proportion of the adolescent population in Malaysia as a whole. Secondly, the cross-sectional study design used in this survey does not permit the cause and effect to be shown from the risk factors for IA among adolescents. Thirdly, the definite diagnostic criterion of IA is still lacking globally, and the use of the Malay version of Young’s internet Addiction Test is based on the DSM-IV addiction criteria, which in this study has set a cutoff point that may differ from other countries. There are also different assessment tools available that yield varying results accordingly. Considering the limitations listed, recent findings should be interpreted cautiously. Despite that, this study carries an equal weight of benefits for future research and intervention planning.

## Conclusions

This study contributes significant insights into the heightened prevalence of IA among adolescents in Malacca State, particularly during the COVID-19 pandemic. The findings underscore the concurrent presence of depression, anxiety, and stress among this demographic, emphasizing the interconnected nature of mental health challenges during these challenging times. Furthermore, the study reinforces the pivotal role of smartphone usage, encompassing factors such as frequency, duration, and late-night usage, in influencing IA patterns among adolescents. The identification of these associations provides a foundation for the implementation of early education and preventive strategies tailored to address the nuanced challenges faced by adolescents. The importance of conducting advanced research is emphasized, which calls for the implementation of large-scale prospective studies to examine the continued existence of IA and related psychological distress in the aftermath of the pandemic, thereby gaining significant knowledge regarding the enduring consequences.

## Supplemental Information

10.7717/peerj.17489/supp-1 Supplemental Information 1 Raw data

## Additional Information and Declarations

### Data Availability

The following information was supplied regarding data availability: The raw data is available in the Supplemental File.
